# Decrease in low-density lipoprotein cholesterol is associated with an increased risk of mortality in patients with diabetes mellitus

**DOI:** 10.3389/fcvm.2025.1549517

**Published:** 2025-05-02

**Authors:** Yun Gi Kim, Joo Hee Jeong, Kyung-Do Han, Seung-Young Roh, Chang-Ok Seo, Yeji Kim, Hyoung Seok Lee, Jaemin Shim, Young-Hoon Kim, Jong-Il Choi

**Affiliations:** ^1^ Division of Cardiology, Department of Internal Medicine, Korea University College of Medicine and Korea University Anam Hospital, Seoul, Republic of Korea; ^2^ Department of Statistics and Actuarial Science, Soongsil University, Seoul, Republic of Korea; ^3^ Division of Cardiology, Department of Internal Medicine, Korea University College of Medicine and Korea University Guro Hospital, Seoul, Republic of Korea

**Keywords:** cholesterol, LDL-cholesterol, diabetes mellitus, sudden cardiac arrest, all-cause mortality

## Abstract

**Introduction:**

Research on the effect of low-density lipoprotein (LDL)-cholesterol levels and its serial change on all-cause mortality is limited. This study investigated serial change in LDL-cholesterol and its association with all-cause mortality or sudden cardiac arrest (SCA) in patients with diabetes mellitus.

**Methods:**

Data was obtained from the nationwide health insurance database of South Korea. Patients with diabetes mellitus who underwent health screening between 2009 and 2012 and those with 4-year follow-up health screening data were included. Patients were further stratified by statin use and change in LDL-cholesterol levels during this 4-year interval. The primary and secondary outcomes were all-cause mortality and SCA, respectively. Outcomes were followed up from the day of health screening till December 2018. Kaplan–Meier analysis and the Cox-proportional hazards model were used to evaluate associations between LDL-cholesterol changes, all-cause mortality, and SCA.

**Results and Discussion:**

A total of 1,329,982 patients were included, including 532,260 patients who did not receive statin therapy (non-statin users). Compared to statin users, non-statin users had a higher incidence of all-cause mortality (incidence rate 13.9–16.4 per 1,000 person-years) and SCA (1.6–1.9). Among non-statin users, patients with decreased LDL-cholesterol had the highest risk of all-cause mortality (adjusted hazard ratio 1.26, 95% confidence interval 1.21–1.31, *P* < 0.001) and SCA (1.21, 1.10–1.34, *P* < 0.001). Thus, in patients with diabetes mellitus not receiving statin therapy, a decrease in LDL-cholesterol may be a surrogate marker for all-cause mortality and SCA.

## Introduction

The association between low-density lipoprotein (LDL)-cholesterol levels and mortality is not clear, and it is interlinked with various comorbid cardiovascular conditions and lipid-lowering therapy. Observational studies report a U-shaped association between LDL-cholesterol levels and risk of all-cause mortality, with the lowest and highest LDL-cholesterol levels carrying greater risks of all-cause mortality (1, 2). The risk of sudden cardiac arrest (SCA) is also higher in people with the lowest and highest LDL-cholesterol levels (3). Various trials have consistently demonstrated that lowering LDL-cholesterol with statins, ezetimibe, proprotein convertase subtilisin/kexin type 9 (PCSK-9), or a combination thereof, reduced the risk of major adverse cardiovascular events, especially in people with atherosclerotic cardiovascular disease (4–7). However, the effect of lipid-lowering treatment on all-cause mortality is unclear. Despite a significant reduction in all-cause mortality in the rosuvastatin arm of the Justification for the Use of Statins in Prevention: an Intervention Trial Evaluating Rosuvastatin (JUPITER) trial, the Antihypertensive and Lipid-Lowering Treatment to Prevent Heart Attack (ALLHAT-LLT) trial failed to prove the mortality related benefits of pravastatin in people with or without coronary artery disease (4, 8). A meta-analysis of 11 randomized controlled trials revealed no all-cause mortality benefit from statin treatment in a high-risk primary prevention setting (9). In the Improved Reduction of Outcomes: Vytorin Efficacy International Trial (IMPROVE-IT) and Further Cardiovascular Outcomes Research with PCSK9 Inhibition in Subjects with Elevated Risk (FOURIER) trial, ezetimibe and PCSK-9 inhibitors also failed to reduce all-cause mortality when prescribed along with statins (6, 7).

The risk of atherosclerotic cardiovascular disease and all-cause mortality is significantly increased in patients with diabetes mellitus (10). Although some variations exist, depending on the presence of target organ damage and duration, the presence of diabetes mellitus is a sufficient condition for high or even very high risk cardiovascular events in both American and European guidelines, with most patients receiving statin therapy to mitigate these risks (11, 12). LDL-cholesterol levels can be a surrogate marker for the risk of all-cause mortality, exempting the beneficial effects of statins and optimal target LDL-cholesterol value (1, 2). However, the association between serial changes in LDL-cholesterol levels (ΔLDL-cholesterol) and all-cause mortality has not been fully evaluated. This study aimed to identify associations between serial changes in LDL-cholesterol levels and all-cause mortality or SCA in people with diabetes mellitus by analyzing the data from a population-based cohort with serial measurements of LDL-cholesterol.

## Materials and methods

### Database

The data for this study was derived from the Korean National Health Insurance Service (K-NHIS) database, which includes virtually all the citizens of the Republic of Korea. The government offers a separate medical aid service for those who cannot afford the subscription fee of the K-NHIS, but the same system as that of the K-NHIS stores their medical data. Therefore, the medical data of the K-NHIS can represent the entire population of the Republic of Korea. The database stores all claims using the International Classification of Disease 10th revision (ICD-10) diagnostic codes and the drug prescription records of the entire country. Furthermore, the K-NHIS provides biennial health screening to adult subscribers to detect diseases and enable early-stage treatment. The nationwide health screening includes the following: (1) self-reported questionnaires of lifestyles and behaviors such as alcohol, smoking, and exercise status; (2) physical examinations such as body weight, height, waist circumference, and blood pressure; and (3) laboratory testing of blood samples, including fasting blood glucose and lipid profiles. The lipid profile includes total cholesterol, triglycerides, high-density lipoprotein levels, and LDL-cholesterol levels, and is calculated using the Friedewald formula. Data were accessed for research purpose from March, 2023 to August, 2023. Authors did not have access to information that could identify individual participants during data collection. The Institutional Review Board of the Korea University Medicine Anam Hospital and the official review committee of the K-NHIS approved this study. Written informed consent was waived by the Institutional Review Board of Korea University Medicine Anam Hospital due to the retrospective nature of the study. The study conformed to the principles of the 2013 Declaration of Helsinki and legal regulations throughout the study period.

### Outcome measurements and definition of variables

The main outcome of this study was all-cause mortality. The death of K-NHIS subscribers is automatically reported to the system, resulting in the automatic termination of their subscriber status. The secondary outcome was SCA, which was identified by the ICD-10 codes used at the emergency department, including “cardiac arrest with successful resuscitation (I46.0),” “sudden cardiac arrest (I46.1),” “cardiac arrest, cause unspecified (I46.9),” “ventricular fibrillation and flutter (I49.0),” “instantaneous death (R96.0),” and “death occurring less than 24 h from symptom onset (R96.1).” If SCA was preceded by hemorrhagic stroke, ischemic stroke, asphyxia, suffocation, drowning, anaphylaxis, gastrointestinal bleeding, major trauma, sepsis, lightning strike, electric shock, or burn events within six months, the event was not considered SCA. We also excluded SCA events that occurred within a year of starting clinical follow-up since those events cannot be discriminated i.e., whether it was an actual SCA event or just a repeat claim of an SCA event that happened before the start of clinical follow-up.

Patients were stratified according to the use of statins at baseline and previous health screenings (4 years ago): (i) patients who were not prescribed statins at baseline or previous health screenings (non-statin users); (ii) patients who were not prescribed statins at previous health screenings but were prescribed statins at baseline (new statin users); and (iii) patients who were prescribed statins at baseline and previous health screenings (constant statin users). Patients were further classified by the degree of LDL-cholesterol change during the 4 years: decreased LDL-cholesterol (ΔLDL-cholesterol < −30 mg/dl), stable LDL-cholesterol (−30 ≤ ΔLDL-cholesterol < + 30 mg/dl), and increased LDL-cholesterol (+30 mg/dl < ΔLDL-cholesterol). The coding strategy, outcome measurement, and variable definitions used in this study were validated in our prior studies (3, 13–15). Supplementary Table S1 summarizes the ICD-10 codes used in this study to identify various medical conditions. Definitions of variables are provided in Supplementary Table S2.

### Study population

Data was obtained from the biennial health screening database of K-NHIS from 2002–2018. The screening period was from January 2002 to the day before health screening and was used to identify the participants’ medical history. The follow-up period was from the day of health screening to December 2018. We first screened people who underwent nationwide health screening from 2009–2012. Among these people, we selected those diagnosed with diabetes mellitus during the screening period. Subsequently, we identified people who underwent sequential health screening four years later (2013–2016) and calculated ΔLDL-cholesterol (baseline LDL-cholesterol measured from 2013–2016 – previous LDL-cholesterol measured from 2009–2012). Underage participants (<20 years old) were excluded from the analysis. There was no upper age limit for inclusion in the study. Triglyceride levels equal to or greater than 400 mg/dl were excluded from the study due to the inaccuracy of calculated LDL-cholesterol levels in people with high triglyceride levels. Since all death events and emigrations are automatically reported to the K-NHIS, resulting in the termination of the subscription, no uncensored follow-up losses exist.

### Statistical analysis

Categorical variables are expressed as numbers and percentages and compared using the Chi-square test or Fisher’s exact test, as appropriate. Continuous variables are expressed as mean ± standard deviation and compared using the Student’s *t*-test or one-way analysis of variance. Non-normally distributed continuous variables were compared using the Mann–Whitney U test. To assess the effect size of continuous variables in one-way analysis of variance, Cohen’s f was calculated, with a value of 0.10 representing a small effect, 0.25 representing a medium effect, and 0.40 representing a large effect. The incidence of all-cause mortality and SCA were described as event numbers per 1,000 person-year follow-up. The cumulative incidence of all-cause mortality and SCA was depicted with Kaplan–Meier survival curve analysis and compared using the log-rank *t*-test. Hazard ratios (HRs) and 95% confidence intervals (CIs) were calculated with Cox-proportional hazards models, with four models: (i) Model 1 was the unadjusted model; (ii) Model 2 was adjusted for age and sex (3), Model 3 was adjusted for age, sex, income, smoking, drinking, regular exercise, hypertension, chronic kidney disease, cardiovascular disease, and previous LDL-cholesterol levels (measured at previous health screenings from 2009–2012), and (4) Model 4 was adjusted for age, sex, income, smoking, drinking, regular exercise, hypertension, chronic kidney disease, cardiovascular disease, fasting glucose level, duration of diabetes mellitus (≥5 years vs. <5 years), use of insulin, use of multiple oral hypoglycemic agents (≥3 drugs vs. <3 drugs), and previous LDL-cholesterol levels. All tests were two-tailed, and a *P*-value ≤ 0.05 was considered statistically significant. All statistical analyses were performed with SAS version 9.2 (SAS Institute, Cary, NC, USA).

## Results

### Baseline characteristics

**Figure 1:**
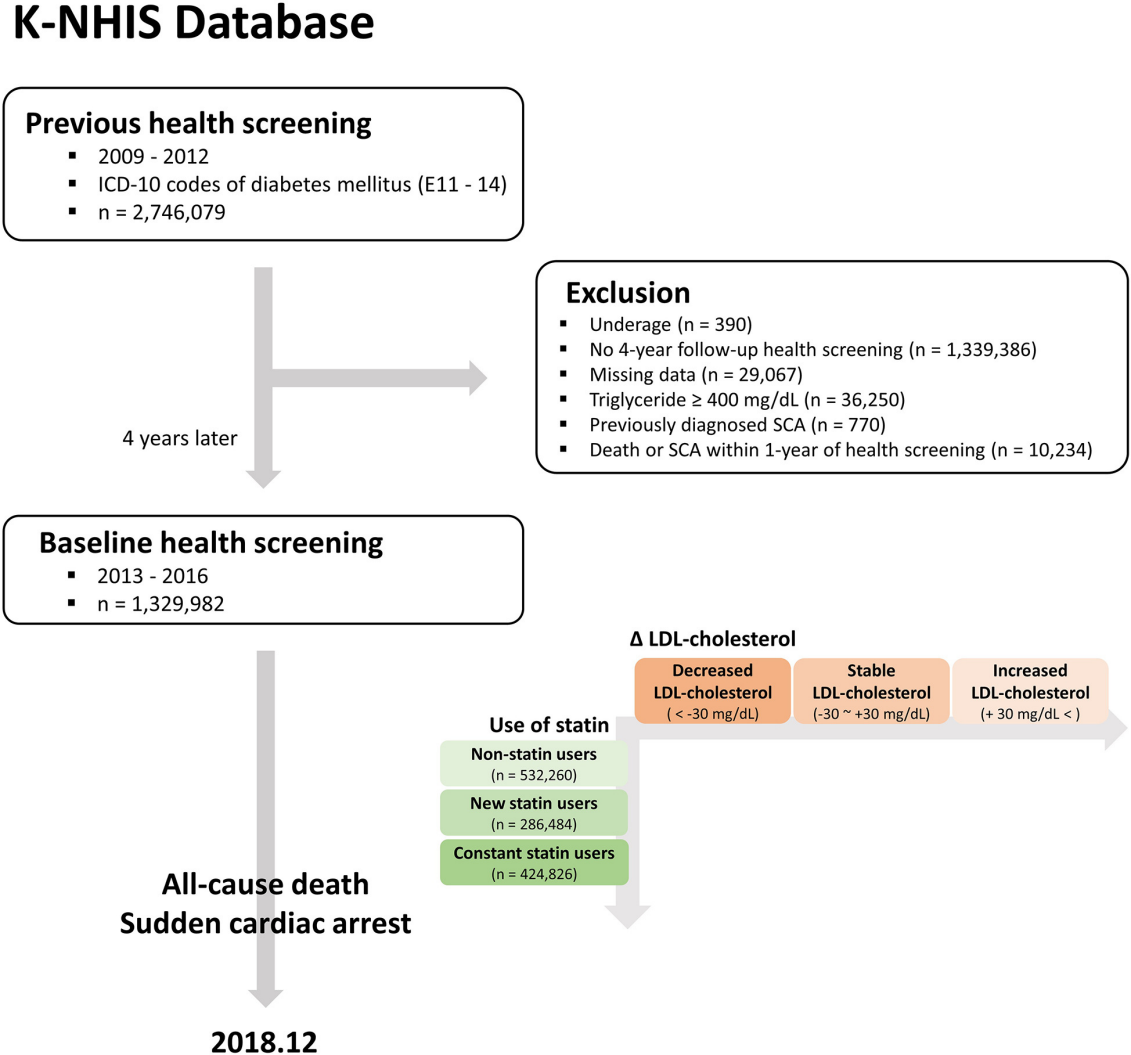
Study flowchart K-NHIS, Korean national health insurance service; ICD-10, international classification of disease, 10th revision; SCA, sudden cardiac arrest; LDL, low-density lipoprotein.

A total of 2,746,079 patients with diabetes mellitus received nationwide health screening between 2009 and 2012 (Figure 1). The following patients were excluded from the analysis: (i) those who were underage (*n* = 390), (ii) those who did not receive health screening 4 years later (*n* = 1,339,386), (iii) those with missing data (*n* = 29,067), (iv) those with triglycerides ≥ 400 mg/dl (*n* = 36,250), (v) those with a previous diagnosis of SCA (*n* = 770), and (vi) those who died or experienced SCA within 1-year of health screening (*n* = 10,234). Among the 1,329,982 patients who received 4-year follow-up health screening, 532,260 were not prescribed statins previously or at baseline (non-statin users), and 286,484 started statin therapy during the 4-year follow-up (new statin users). Consequently, 424,826 patients were prescribed statins at baseline and previous health screenings (constant statin users), and 86,412 were prescribed statins 4 years ago but not at the baseline health screening (stopped statins).

In non-statin users (*n* = 523,260), LDL-cholesterol decreased by more than 30 mg/dl in 80,435 patients during the 4-year follow-up (decreased LDL-cholesterol group), whereas LDL-cholesterol increased by more than 30 mg/dl in 77,074 patients (increased LDL-cholesterol group, Table 1). The change in LDL-cholesterol was 30 mg/dl or less in 374,751 patients (stable LDL-cholesterol). Among the three groups, small differences were found in the baseline characteristics such as age (61.5 ± 11.2 vs. 61.2 ± 11.3 vs. 60.0 ± 11.6 years, *P* < 0.001), hypertension (58.5% vs. 56.3% vs. 55.0%, *P* < 0.001), chronic kidney disease (10.2% vs. 9.1% vs. 10.0%, *P* < 0.001), and cardiovascular disease (4.6% vs. 3.9% vs. 3.8%, *P* < 0.001). Although the decreased LDL-cholesterol group had a lower proportion of prolonged diabetes mellitus (55.0% vs. 58.6% vs. 55.5%. *P* < 0.001), they had more frequent insulin use (8.8% vs. 8.4% vs. 8.4%, *P* < 0.001) or were on multiple oral hypoglycemic agents (24.3% vs. 23.4% vs. 21.6%, *P* < 0.001).

**Table 1:** Baseline characteristics of non-statin users.

Variables	Decreased LDL-cholesterol	Stable LDL-cholesterol	Increased LDL-cholesterol	*P*-value	Cohen’s f
	(*n* = 80,435)	(*n* = 374,751)	(*n* = 77,074)		
Previous LDL-cholesterol (mg/dl)	149.6 ± 169.5	109.5 ± 28.0	87.4 ± 32.2	<0.001	0.174
Baseline LDL-cholesterol (mg/dl)	90.1 ± 28.7	108.8 ± 27.9	135.9 ± 34.5	<0.001	0.417
Age (years)	61.5 ± 11.2	61.2 ± 11.3	60.0 ± 11.6	<0.001	0.023
Age groups (years)				<0.001	
<40	2,034 (2.5%)	10,560 (2.8%)	2,750 (3.6%)		
40–64	45,949 (57.1%)	215,702 (57.6%)	47,062 (61.1%)		
≥65	32,452 (40.4%)	148,489 (39.6%)	27,262 (35.4%)		
Male sex	51,802 (64.4%)	251,793 (67.2%)	50,579 (65.6%)	<0.001	
Body mass index (kg/m^2^)	24.6 ± 3.3	24.6 ± 3.3	24.7 ± 3.2	0.006	0.012
Waist circumference (cm)	84.9 ± 8.6	84.9 ± 8.5	85.0 ± 8.4	0.891	0.004
Income, lowest quartile	15,836 (19.7%)	71,211 (19.0%)	15,599 (20.2%)	<0.001	
Smoking				<0.001	
Non-smoker	44,542 (55.4%)	202,483 (54.0%)	41,169 (53.4%)		
Ex-smoker	17,911 (22.3%)	89,902 (24.0%)	17,101 (22.2%)		
Current smoker	17,982 (22.4%)	82,366 (22.0%)	18,804 (24.4%)		
Drinking				<0.001	
Non-drinker	46,384 (57.7%)	210,934 (56.3%)	42,255 (54.8%)		
Mild drinker	26,377 (32.8%)	130,980 (35.0%)	26,863 (34.9%)		
Heavy drinker	7,674 (9.5%)	32,837 (8.8%)	7,956 (10.3%)		
Regular exercise	19,054 (23.7%)	92,701 (24.7%)	17,539 (22.8%)	<0.001	
Hypertension	47,016 (58.5%)	210,864 (56.3%)	42,416 (55.0%)	<0.001	
Chronic kidney disease	8,191 (10.2%)	34,197 (9.1%)	7,670 (10.0%)	<0.001	
Cardiovascular disease	3,734 (4.6%)	14,451 (3.9%)	2,908 (3.8%)	<0.001	
Prolonged diabetes mellitus (duration ≥5 years)	44,213 (55.0%)	219,548 (58.6%)	42,755 (55.5%)	<0.001	
Insulin use	7,078 (8.8%)	31,313 (8.4%)	6,497 (8.4%)	<0.001	
Multiple oral hypoglycemic agents (≥3)	19,576 (24.3%)	87,856 (23.4%)	16,656 (21.6%)	<0.001	
Systolic blood pressure (mmHg)	128.6 ± 15.1	128.1 ± 15.0	128.8 ± 15.2	<0.001	0.019
Diastolic blood pressure (mmHg)	77.8 ± 9.8	77.7 ± 9.8	78.6 ± 9.8	<0.001	0.035
Fasting glucose (mg/dl)	144.0 ± 48.4	147.3 ± 47.6	155.1 ± 52.9	<0.001	0.065
Total cholesterol (mg/dl)	173.1 ± 32.7	186.5 ± 32.3	214.8 ± 37.2	<0.001	0.349
HDL-cholesterol (mg/dl)	50.5 ± 15.5	50.0 ± 13.8	50.2 ± 13.3	<0.001	0.013
Triglycerides (mg/dl)^a^	141 (95–211)	123 (87–175)	131 (95–182)	<0.001	

Parameters obtained at the time of follow-up LDL-cholesterol measurement were used for the baseline demographics.

HDL, high-density lipoprotein; LDL, low-density lipoprotein.

^a^ Triglycerides were described as medians and quartiles.

Compared with constant or new statin users, non-statin users were younger and mostly males (Supplementary Tables S3–S4). Non-statin users also had lower body mass indexes and waist circumferences and a lower proportion of cardiovascular comorbidities, including hypertension, chronic kidney disease, cardiovascular disease, prolonged diabetes mellitus, and the use of insulin or multiple oral hypoglycemic agents. In contrast, the proportion of current smokers and heavy drinkers was higher among the non-statin users.

### Primary outcome: all-cause mortality

**Figure 2:**
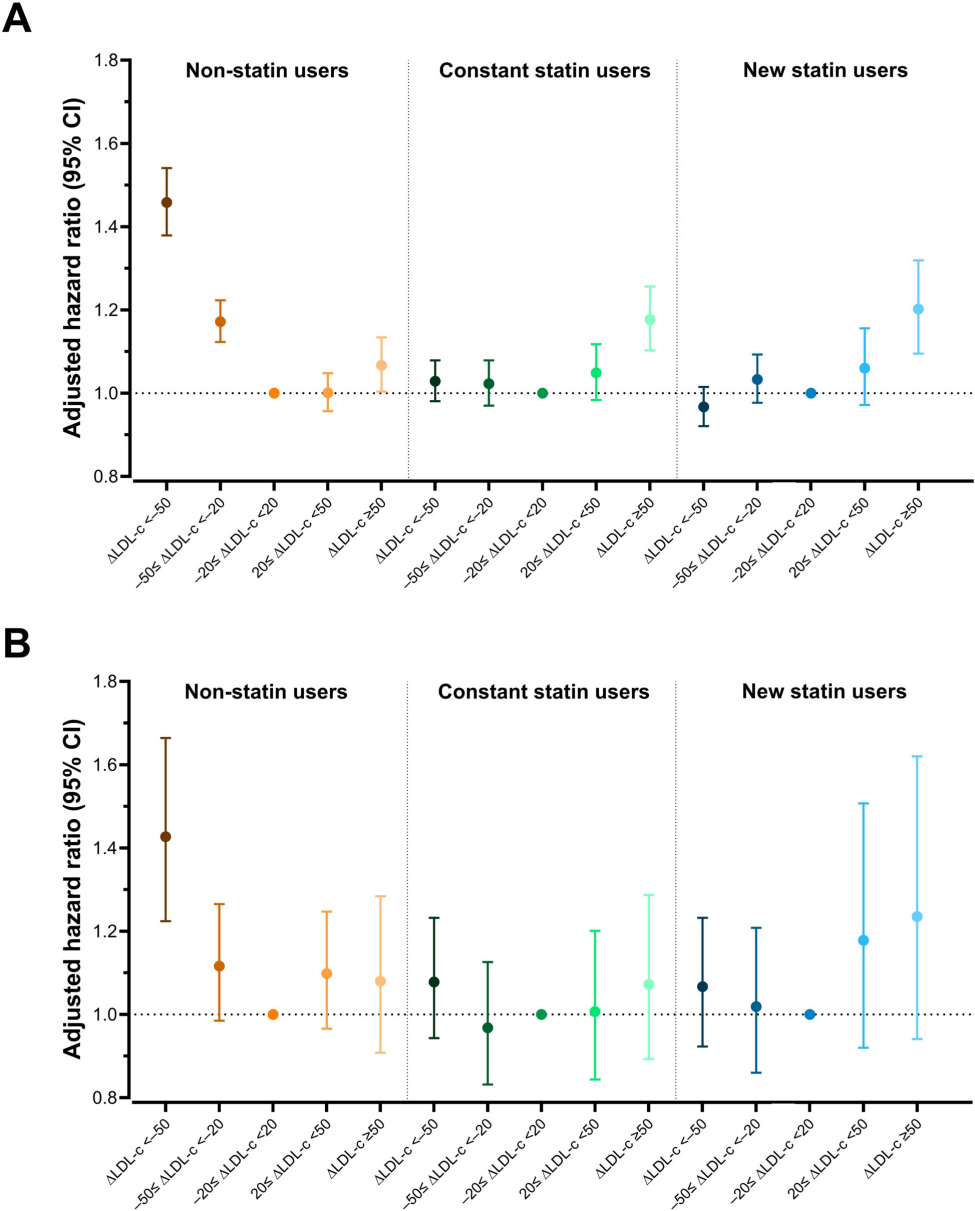
LDL-cholesterol change and risk of the outcomes **(A)** all-cause death, **(B)** sudden cardiac arrest. The stable LDL-cholesterol subgroup was the reference in each group. Hazard ratios were adjusted for age, sex, body mass index, income, smoking status, alcohol consumption status, regular exercise, hypertension, chronic kidney disease, cardiovascular disease, previous LDL-cholesterol levels, fasting glucose, duration of diabetes mellitus, use of insulin, and use of multiple (≥3) oral hypoglycemic agents. ΔLDL-c, changes in low-density lipoprotein cholesterol; CI, confidence interval.

According to statin usage, mortality was highest in non-statin users, followed by constant and new statin users. In patients who received statins (constant and new statin users), the mortality rate was highest in the increased LDL-cholesterol group (incidence = 11.6 and 12.3 for constant and new statin users, respectively, Supplementary Tables S5–S6). In constant statin users, adjustment of demographic factors, social habits, cardiovascular comorbidities, and previous LDL-cholesterol levels revealed the highest risk of mortality in the increased LDL-cholesterol group compared to the stable LDL-cholesterol group (adjusted HR = 1.11, 95% CI 1.06–1.16, *P* < 0.001), followed by that in the decreased LDL-cholesterol group (adjusted HR = 1.03, 95% CI 0.99–1.07, *P* = 0.20, Supplementary Table S5). Furthermore, the risk of mortality was magnified in patients with LDL-cholesterol increases of more than 50 mg/dl (adjusted HR = 1.18, 95% CI 1.10–1.26, *P* < 0.001, Supplementary Table S7, Figure 2A).

Conversely, in non-statin users, the mortality rate was highest in the decreased LDL-cholesterol group (incidence = 16.4), followed by the increased LDL-cholesterol group (incidence = 14.6), and stable LDL-cholesterol group (incidence = 13.9, Table 2). Adjustment of covariates revealed a 25.9% increased risk of mortality in the decreased LDL-cholesterol group (Model 4, adjusted HR = 1.26, 95% CI 1.21–1.31, *P* < 0.001, Table 2). A decrease in LDL-cholesterol of more than 50 mg/dl revealed a 45.8% increased risk of mortality (Model 4, adjusted HR = 1.46, 95% CI 1.38–1.54, *P* < 0.001, Supplementary Table S7, Figure 2A).

**Table 2:** Impact of LDL-cholesterol change in non-statin users.

Variables	*n*	Events	Duration (person-years)	Incidence	Hazard ratio (95% confidence interval)				*P-*value^a^
					Model 1	Model 2	Model 3	Model 4	
All-cause mortality									
Decreased LDL-cholesterol	80,435	4,169	254,588	16.4	1.175 (1.136–1.216)	1.194 (1.154–1.235)	1.274 (1.228–1.321)	1.259 (1.214–1.306)	<0.001
Stable LDL-cholesterol	374,751	16,538	1,187,480	13.9	1 (reference)	1 (reference)	1 (reference)	1 (reference)	
Increased LDL-cholesterol	77,074	3,603	246,811	14.6	1.044 (1.007–1.083)	1.153 (1.112–1.195)	1.026 (0.987–1.067)	1.028 (0.989–1.069)	0.163
Sudden cardiac arrest									
Decreased LDL-cholesterol	80,435	475	254,498	1.9	1.199 (1.084–1.326)	1.226 (1.108–1.356)	1.202 (1.085–1.332)	1.213 (1.095–1.344)	<0.001
Stable LDL-cholesterol	374,751	1,847	1,187,192	1.6	1 (reference)	1 (reference)	1 (reference)	1 (reference)	
Increased LDL-cholesterol	77,074	428	246,748	1.7	1.112 (1.001–1.235)	1.210 (1.089–1.344)	1.112 (0.997–1.241)	1.093 (0.980–1.219)	0.110

Incidence is per 1,000 person*year follow-up.

LDL, low-density lipoprotein.

Model 1: Unadjusted.

Model 2: Adjusted for age and sex.

Model 3: Adjusted for age, sex, body mass index, income, smoking status, alcohol consumption status, regular exercise, hypertension, chronic kidney disease, cardiovascular disease, and previous LDL-cholesterol levels.

Model 4: Adjusted for age, sex, body mass index, income, smoking status, alcohol consumption status, regular exercise, hypertension, chronic kidney disease, cardiovascular disease, previous LDL-cholesterol levels, fasting glucose, duration of diabetes mellitus, use of insulin, and use of multiple (≥3) oral hypoglycemic agents.

^a^ *P*-value is described for the adjusted hazard ratio in Model 4.

### Secondary outcome: sudden cardiac arrest

**Figure 3:**
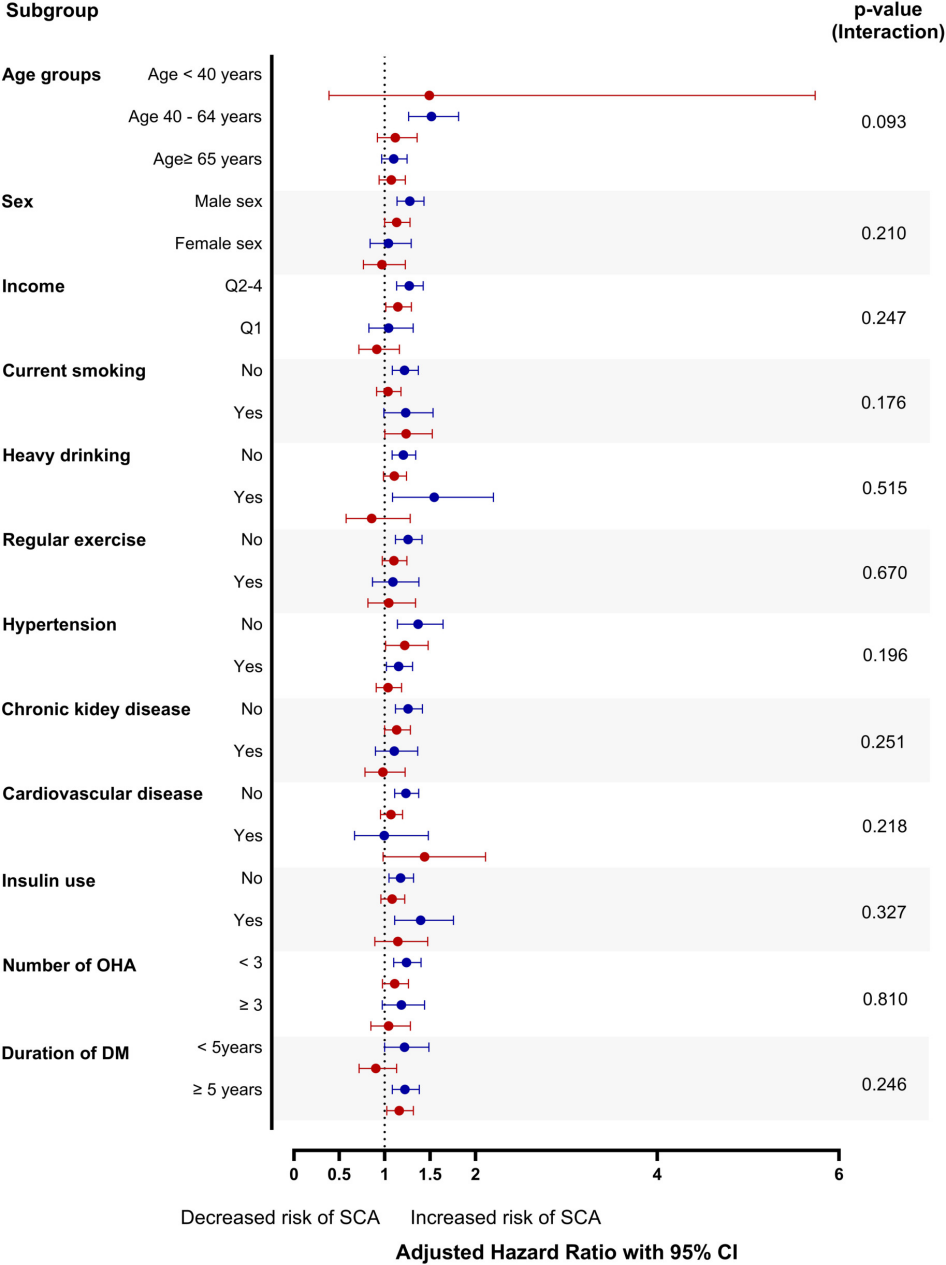
Subgroup analysis for SCA in non-statin users the stable LDL-cholesterol subgroup was the reference in each group. CI, confidence interval; DM, diabetes mellitus; LDL, low-density lipoprotein; OHA, oral hypoglycemic agent; Q, quartile (Q1 indicates lowest income level); SCA, sudden cardiac arrest.

In patients who were prescribed statins (constant and new statin users), the incidence of SCA was highest in the increased LDL-cholesterol group (incidence = 1.5 and 1.5 in constant and new statin users, respectively), while the decreased LDL-cholesterol group had the highest SCA incidence in non-statin users (incidence rate = 1.9, Table 2, Supplementary Tables S5–S6). Adjustment of multiple covariates revealed a 21.3% increased risk of SCA in the decreased LDL group in non-statin users (adjusted HR = 1.21, 95% CI 1.10–1.34, *P* < 0.001, Table 2). This phenomenon was magnified with a substantial change in LDL-cholesterol (ΔLDL-cholesterol < −50 mg/dl), which resulted in a 42.7% increase in SCA risk in the decreased LDL-cholesterol group (adjusted HR = 1.43, 95% CI 1.22–1.66, *P* < 0.001, Supplementary Table S8, Figure 2B). Non-statin users were further divided into subgroups according to age, sex, income, smoking status, drinking status, regular exercise, and cardiovascular conditions and comorbidities (Figure 3). None of the subgroups showed significant interactions.

## Discussion

This study investigated the effect of LDL-cholesterol change on mortality in patients with diabetes mellitus and further stratified the analysis according to statin usage during a 4-year follow-up. The major findings from this study are as follows: (i) In patients who received statin therapy, an increase in LDL-cholesterol during follow-up was associated with an increased risk of mortality and SCA, whereas a decrease in LDL-cholesterol did not show any significant association with clinical outcomes; (ii) Although non-statin users were younger and with fewer cardiovascular comorbidities compared with statin users (constant and new statin users), they had a higher incidence of mortality and SCA; (iii) In non-statin users, no association was found between increases in LDL-cholesterol levels and risk of all-cause mortality or SCA. However, a decrease in LDL-cholesterol levels during follow-up was associated with a paradoxically increased risk of all-cause mortality.

The current study was based on 1.24 million patients with diabetes mellitus and included baseline and follow-up LDL-cholesterol levels. The total follow-up duration was 3.87 million person-years. Another strength of the study was the lack of uncensored follow-up losses. Owing to the intrinsic nature of the K-NHIS database, no death event would be left unnoticed by the system. The coding strategy was validated in our prior publications (3, 13, 16, 17).

### LDL-cholesterol and all-cause mortality

The association between LDL-cholesterol levels and all-cause mortality was U-shaped, with the highest and lowest LDL levels having greater risk (1, 2). Our study suggests that a decrease in LDL-cholesterol levels during a given period in patients with diabetes mellitus who are non-statin users may be associated with increased mortality. This may be due to the malnourished condition that emerges over time with the decrease in LDL-cholesterol. Body mass index, hemoglobin, and total cholesterol are reliable markers for malnutrition, which is a significant risk factor for mortality (18, 19). Medical diseases that can cause malnutrition, such as cancers, liver and kidney disease, chronic obstructive pulmonary disease, or hyperthyroidism may have a direct impact on all-cause mortality. Additionally, a decrease in serum cholesterol may be related to a weakening immune system. Serum cholesterol has been reported to have a protective role in the immune system by increasing lymphocytes or binding to endotoxins (20, 21). A diminished immune response mediated by lower LDL-cholesterol may predispose patients to systemic infections or a severe illness that leads to death. Although this study suggests a significant association, whether a decline in LDL-cholesterol directly affects all-cause mortality or is just a marker for malnutrition or an underlying medical condition remains unclear. Since we adjusted for the influence of body mass index, a strong marker of malnutrition, the association observed in this study may have an additional linking mechanism other than malnutrition. At least, a decrease in LDL-cholesterol levels can be considered a surrogate marker for increased mortality in patients with diabetes mellitus who are not on statin therapy.

Reduction of cardiovascular death due to statin use in diabetic people with elevated LDL-cholesterol is well established. However, whether it can reduce all-cause death remains controversial. An observational study from Iceland reported that statin use was associated with 16% and 30% reduced risks of cardiovascular and all-cause mortality, respectively (22). A recent meta-analysis revealed that statin use in patients with diabetes mellitus was associated with a reduced risk of cardiovascular events but not with all-cause mortality (23). Statin use was associated with a numerically lower risk of all-cause mortality (HR 0.89 and 0.97 for primary and secondary prevention, respectively), but this was not statistically significant. Most of the included randomized clinical trials were performed in the late 1990s and early 2000s, and it is questionable whether statins will have any all-cause mortality benefits in the contemporary era with more advanced medications for diabetes mellitus, such as sodium-glucose cotransporter-2 inhibitors.

Recent guidelines recommend more intense lowering of LDL-cholesterol levels in patients with diabetes mellitus (11, 12). We did not observe any all-cause mortality benefit in patients with diabetes mellitus who had a >50 mg/dl reduction in LDL-cholesterol in both non-statin and statin users. However, a greater than 50 mg/dl increase in LDL-cholesterol level was associated with an increased risk of all-cause mortality regardless of statin use. Although lowering LDL-cholesterol by statin therapy can decrease the incidence of various cardiovascular events, its effect on all-cause mortality needs further validation.

### LDL-cholesterol and sudden cardiac arrest

The association of LDL-cholesterol changes and SCA was similar to that of all-cause mortality but showed weaker association. In non-statin users, the risk of SCA was also highest in those who experienced a significant decline in LDL-cholesterol levels. However, no significant increase of SCA risk was observed in statins users who had increased LDL-cholesterol. This weaker association could be due to fewer SCA events compared to all-cause mortality. For instance, the increased LDL-cholesterol group in new statin users had a higher incidence of SCA compared to the decreased or stable LDL-cholesterol group. However, no statistical significance was found due to small event size. The association between statin use, LDL-cholesterol level, and the risk of SCA in patients with diabetes mellitus is not fully understood and is an area for future research.

An interesting finding is the differential effect of confounders according to the LDL-cholesterol change in non-statin users, for both all-cause mortality and SCA. In the decreased LDL-cholesterol group, adjustment of covariates accentuates the association between LDL-cholesterol changes and outcome. However, adjustment of covariates in the increased LDL-cholesterol group minimizes the association between LDL-cholesterol changes and outcomes, especially after adjusting of comorbidities and lifestyle habits (Model 3). This finding implies that elevated mortality or SCA incidence in increased LDL-cholesterol is significantly confounded by comorbid conditions such as obesity or metabolic diseases, whereas decreased LDL-cholesterol has a more robust and distinct link to the outcomes, which is not explained by metabolic risk factors.

### Limitations

This study had some limitations. First, unmeasured confounding factors can exist in our study because this is an intrinsic limitation of retrospective analyses. Although we adjusted for multiple covariates, including smoking, alcohol, past medical history, and diabetes severity, unidentified confounders in the claim database could have affected our results. Second, although medications for diabetes mellitus, its duration, and fasting blood glucose were adjusted for analysis, HbA1c levels were unadjusted since the data was unavailable in the K-NHIS system. Third, the LDL-cholesterol level in our study was calculated using total cholesterol, triglycerides, and high-density lipoprotein cholesterol. Calculated cholesterol can be inaccurate with high triglyceride levels, and therefore, we excluded people with triglycerides ≥ 400 mg/dl. Fourth, we classified patients according to statin use during serial health screening, and other lipid-lowering therapies, such as ezetimibe, fibrates, or PCSK-9 inhibitors, were not considered. Lastly, our cohort consists of East Asians, and therefore, extrapolation of our results to other population groups should be done with caution.

## Conclusions

In patients with diabetes mellitus not on statin therapy, decreases in LDL-cholesterol levels were associated with an increased risk of all-cause mortality and SCA. In patients with diabetes mellitus on statin therapy, a decrease in LDL-cholesterol levels did not influence all-cause mortality and SCA. Thus, a decline in LDL-cholesterol levels in patients with diabetes mellitus who are not on statins can be considered a surrogate marker for all-cause mortality and SCA.

## Data Availability

The original contributions presented in the study are included in the article/Supplementary Material, further inquiries can be directed to the corresponding authors.
