# Supplementary material for: Decrease in low-density lipoprotein cholesterol is associated with an increased risk of mortality in patients with diabetes mellitus
Source: Front Cardiovasc Med. 2025 May 2;12:1549517. doi: 10.3389/fcvm.2025.1549517 (PMC12105105; doi:10.3389/fcvm.2025.1549517)
Supplement: Supplementary file 1 [file Table1.docx]

**Supplementary Materials**

**Supplementary Table 1. ICD-10 codes for diagnosis**

| **Diseases** | **ICD-10 Codes** |
| --- | --- |
| **Sudden cardiac arrest** | I46.0, I46.1, I46.9, I49.0, R96.0, R96.1 |
| Cardiac arrest with successful resuscitation | I46.0 |
| Sudden cardiac arrest | I46.1 |
| Cardiac arrest, cause unspecified | I46.9 |
| Ventricular fibrillation and flutter | I49.0 |
| Instantaneous death | R96.0 |
| Death occurring less than 24 hours from symptom onset | R96.1 |
| **Hypertension** | I10 – I13, I15 (all sub-codes) |
| **Diabetes mellitus (Type 2)** | E11 – E14 (all sub-codes) |
| **Dyslipidemia** | I78 (all sub-codes) |

ICD-10: International Classification of Diseases, tenth edition.

**Supplementary Table 2. Definitions of variables**

|  | **Definition** |
| --- | --- |
| **Smoking** | |
| Non-smoker | <100 cigarettes in the lifetime |
| Ex-smoker | ≥100 cigarettes in the lifetime, but had not smoked within 1 month of the health check-up in 2009 |
| Current smoker | ≥100 cigarettes in the lifetime, and continued smoking within 1 month of the health check-up in 2009 |
| **Alcohol consumption** |  |
| Non-drinker | Those who consumed 0 g of alcohol per week |
| Mild drinker | Those who consumed < 210 g of alcohol per week |
| Heavy drinker | Those who consumed ≥ 210 g of alcohol per week |
| **Hypertension** | |
| Non-hypertensive | SBP < 120, DBP < 80, and no diagnostic codes for hypertension |
| Hypertension | SBP ≥ 140 or DBP ≥ 90 or diagnostic codes for hypertension |
| **Regular exercise** | Those who had one or more weekly sessions of high (such as running, climbing, intense bicycle activities) or moderate physical activity (such as walking fast, tennis, or moderate bicycle activities). |
| Cardiovascular disease | Previous diagnosis of myocardial infarction or stroke |
| Chronic kidney disease | Estimated glomerular filtration rate < 60ml/min/1.73 m2 by the Modification of Diet in Renal Disease equation |

DBP: Diastolic blood pressure, mmHg; SBP: Systolic blood pressure, mmHg.

**Supplementary Table 3. Baseline characteristics in constant statin users**

|  | **Decreased LDL-cholesterol** (n=126,433) | **Stable LDL-cholesterol** (n=240,778) | **Increased LDL-cholesterol** (n=57,615) | ***P*-value** |
| --- | --- | --- | --- | --- |
| Previous LDL-cholesterol (mg/dL) | 145.3 ± 123.6 | 87.3 ± 29.2 | 73.1 ± 31.3 | <.001 |
| Baseline LDL-cholesterol (mg/dL) | 75.5 ± 26.5 | 85.5 ± 28.9 | 128.9 ± 40.8 | <.001 |
| Age (years) | 63.3 ± 9.5 | 64.3 ± 9.39 | 63.0 ± 9.6 | <.001 |
| Age groups (years) |  |  |  | <.001 |
| <40 | 822 (0.7%) | 1,190 (0.5%) | 383 (0.7%) |  |
| 40-64 | 69,301 (54.8%) | 121,282 (50.4%) | 32,364 (56.2%) |  |
| ≥65 | 56,310 (44.5%) | 118,306 (49.1%) | 24,868 (43.2%) |  |
| Male sex | 58,009 (45.9%) | 118,846 (49.4%) | 27,965 (48.5%) | <.001 |
| Body mass index (kg/m^2^) | 25.3 ± 3.3 | 25.3 ± 3.3 | 25.3 ± 3.2 | 0.221 |
| Waist circumference (cm) | 86.0 ± 8.7 | 86.3 ± 8.6 | 86.2 ± 8.6 | <.001 |
| Income, Lowest Q1 | 25,788 (20.4%) | 46,970 (19.5%) | 11,781 (20.5%) | <.001 |
| Smoking |  |  |  | <.001 |
| Non-smoker | 83,003 (65.7%) | 153,562 (63.8%) | 36,580 (63.5%) |  |
| Ex-smoker | 23,968 (19.0%) | 51,002 (21.2%) | 11,202 (19.4%) |  |
| Current smoker | 19,462 (15.4%) | 36,214 (15.0%) | 9,833 (17.1%) |  |
| Drinking |  |  |  | <.001 |
| Non-drinker | 89,005 (70.4%) | 168,034 (69.8%) | 39,233 (68.1%) |  |
| Mild drinker | 30,724 (24.3%) | 60,048 (24.9%) | 14,718 (25.6%) |  |
| Heavy drinker | 6,704 (5.3%) | 12,696 (5.3%) | 3,664 (6.4%) |  |
| Regular exercise | 29,285 (23.2%) | 57,808 (24.0%) | 13,136 (22.8%) | <.001 |
| Hypertension | 89,121 (70.5%) | 178,545 (74.2%) | 41,752 (72.5%) | <.001 |
| Chronic kidney disease | 18,858 (14.9%) | 39,903 (16.6%) | 10,423 (18.1%) | <.001 |
| Cardiovascular disease | 12,102 (9.6%) | 24,967(10.4%) | 5,624 (9.8%) | <.001 |
| Prolonged diabetes mellitus (duration ≥5 years) | 84,188 (66.6%) | 188,814 (78.4%) | 44,467 (77.2%) | <.001 |
| Insulin use | 16,164 (12.8%) | 34,862 (14.5%) | 8,920 (15.5%) | <.001 |
| Multiple oral hypoglycemic agents (≥3) | 44,843 (35.5%) | 86,153 (35.8%) | 20,849 (36.2%) | 0.010 |
| Systolic blood pressure (mmHg) | 127.5 ± 14.8 | 128.0 ± 14.8 | 128.7 ± 15.2 | <.001 |
| Diastolic blood pressure (mmHg) | 76.3 ± 9.4 | 76.2 ± 9.5 | 77.2 ± 9.6 | <.001 |
| Fasting glucose (mg/dL) | 135.4 ± 41.2 | 136.6 ± 41.8 | 143.0 ± 47.8 | <.001 |
| Total cholesterol (mg/dL) | 156.0 ± 30.7 | 164.1 ± 34.2 | 210.8 ± 43.2 | <.001 |
| HDL-cholesterol (mg/dL) | 51.3 ± 13.8 | 50.6 ± 13.1 | 50.9 ± 13.4 | <.001 |
| Triglycerides (mg/dL)^a^ | 128 [92 - 182] | 125 [90 - 174] | 143 [104 - 198] | <.001 |

^a^Triglycerides were described in medians and quartiles.

LDL, low-density lipoprotein, HDL, high-density lipoprotein.

**Supplementary Table 4. Baseline characteristics in new statin users**

|  | **Decreased LDL-cholesterol** (n=149,801) | **Stable LDL-cholesterol** (n=108,611) | **Increased LDL-cholesterol** (n=28,072) | ***P*-value** |
| --- | --- | --- | --- | --- |
| Previous LDL-cholesterol (mg/dL) | 141.1 ± 103.2 | 111.5 ± 32.0 | 93.5 ± 37.2 | <.001 |
| Baseline LDL-cholesterol (mg/dL) | 72.8 ± 25.0 | 107.6 ± 34.7 | 146.9 ± 41.7 | <.001 |
| Age (years) | 61.4 ± 10.5 | 61.4 ± 10.6 | 60.7 ± 10.7 | <.001 |
| Age groups (years) |  |  |  | <.001 |
| <40 | 2,675 (1.8%) | 2,028 (1.9%) | 582 (2.1%) |  |
| 40-64 | 89,485 (59.7%) | 64,267 (59.2%) | 17,227 (61.4%) |  |
| ≥65 | 57,641 (38.5%) | 42,316 (39.0%) | 10,263 (36.6%) |  |
| Male sex | 83,714 (55.9%) | 64,282 (59.2%) | 16,070 (57.3%) | <.001 |
| Body mass index (kg/m^2^) | 25.2 ± 3.3 | 25.1 ± 3.3 | 24.9 ± 3.2 | <.001 |
| Waist circumference (cm) | 85.8 ± 8.6 | 85.9 ± 8.5 | 85.4 ± 8.4 | <.001 |
| Income, Lowest Q1 | 29,814 (19.9%) | 21,609 (19.9%) | 5,751 (20.5%) | 0.065 |
| Smoking |  |  |  | <.001 |
| Non-smoker | 88,606 (59.2%) | 62,102 (57.2%) | 16,190 (57.7%) |  |
| Ex-smoker | 33,201 (22.2%) | 24,439 (22.5%) | 5,963 (21.2%) |  |
| Current smoker | 27,994 (18.7%) | 22,070 (20.3%) | 5,919 (21.1%) |  |
| Drinking |  |  |  | <.001 |
| Non-drinker | 96,686 (64.5%) | 66,612 (61.3%) | 17,044 (60.7%) |  |
| Mild drinker | 43,787 (29.2%) | 33,466 (30.8%) | 8,505 (30.3%) |  |
| Heavy drinker | 9,328 (6.2%) | 8,533 (7.9%) | 2,523 (9.0%) |  |
| Regular exercise | 34,959 (23.3%) | 25,189 (23.2%) | 6,231(22.2%) | <.001 |
| Hypertension | 97,665(65.2%) | 69,692(64.2%) | 17,479(62.3%) | <.001 |
| Chronic kidney disease | 17,962 (12.0%) | 13,526 (12.5%) | 3,686 (13.1%) | <.001 |
| Cardiovascular disease | 14,387 (9.6%) | 9,171 (8.4%) | 2,085 (7.4%) | <.001 |
| Prolonged diabetes mellitus (duration ≥5 years) | 88,756 (59.3%) | 66,673 (61.4%) | 16,324 (58.2%) | <.001 |
| Insulin use | 17,561 (11.7%) | 13,556 (12.5%) | 3,403 (12.1%) | <.001 |
| Multiple oral hypoglycemic agents (≥3) | 55,042 (36.7%) | 38,459 (35.4%) | 9,373 (33.4%) | <.001 |
| Systolic blood pressure (mmHg) | 127.0 ± 14.6 | 127.9 ± 14.9 | 128.6 ± 15.4 | <.001 |
| Diastolic blood pressure (mmHg) | 76.6 ± 9.5 | 77.4 ± 9.6 | 78.2 ± 9.8 | <.001 |
| Fasting glucose (mg/dL) | 138.8 ± 41.9 | 145.5 ± 47.6 | 153.2 ± 55.0 | <.001 |
| Total cholesterol (mg/dL) | 149.8 ± 30.2 | 186.8 ± 39.9 | 227.9 ± 42.3 | <.001 |
| HDL-cholesterol (mg/dL) | 50.3 ± 13.3 | 49.8 ± 13.6 | 50.4 ± 15.1 | <.001 |
| Triglycerides (mg/dL)^a^ | 116 [83 - 164] | 130 [92 - 186] | 142 [102 - 198] | <.001 |

^a^Triglycerides were described in medians and quartiles.

LDL, low-density lipoprotein, HDL, high-density lipoprotein.

**Supplementary Table 5. Impact of LDL-cholesterol change on the primary and secondary outcomes of constant statin users**

|  | **n** | **Events** | **Duration (person-years)** | **Incidence** | **Hazard ratio (95% confidence interval)** | | | | ***P*-value^a^** |
| --- | --- | --- | --- | --- | --- | --- | --- | --- | --- |
|  |  |  |  |  | **Model 1** | **Model 2** | **Model 3** | **Model 4** |  |
| **Primary outcome** |  |  |  |  |  |  |  |  |  |
| Decreased LDL-cholesterol | 126,433 | 3,968 | 384,793 | 10.3 | 0.909 (0.875 - 0.944) | 1.010 (0.973 - 1.049) | 1.007 (0.969 - 1.048) | 1.026 (0.987 - 1.067) | 0.195 |
| Stable LDL-cholesterol | 240,778 | 8,316 | 733,422 | 11.3 | 1 (Reference) | 1 (Reference) | 1 (Reference) | 1 (Reference) |  |
| Increased LDL-cholesterol | 57,615 | 2,094 | 180,038 | 11.6 | 1.019 (0.972 - 1.069) | 1.162 (1.108- 1.219) | 1.126 (1.073- 1.182) | 1.109 (1.057- 1.163) | <0.001 |
| **Secondary outcome** |  |  |  |  |  |  |  |  |  |
| Decreased LDL-cholesterol | 126,433 | 521 | 384,686 | 1.4 | 0.902 (0.913 - 1.001) | 0.989 (0.891 - 1.098) | 1.001 (0.895 - 1.119) | 1.027 (0.921 - 1.146) | 0.627 |
| Stable LDL-cholesterol | 240,778 | 1,100 | 733,217 | 1.5 | 1 (Reference) | 1 (Reference) | 1 (Reference) | 1 (Reference) |  |
| Increased LDL-cholesterol | 57,615 | 269 | 179,982 | 1.5 | 0.992 (0.868 - 1.133) | 1.098 (0.960 - 1.254) | 1.048 (0.916 - 1.198) | 1.036 (0.907 - 1.185) | 0.601 |

^a^*P*-value is described for the adjusted hazard ratio in Model 4.

Incidence is per 1,000 person*year follow-up.

LDL, low-density lipoprotein.

Model 1: Unadjusted.

Model 2: Adjusted for age and sex.

Model 3: Adjusted for age, sex, body mass index, income, smoking status, alcohol consumption status, regular exercise, hypertension, chronic kidney disease, cardiovascular disease, and previous LDL-cholesterol levels.

Model 4: Adjusted for age, sex, body mass index, income, smoking status, alcohol consumption status, regular exercise, hypertension, chronic kidney disease, cardiovascular disease, previous LDL-cholesterol levels, fasting glucose, duration of diabetes mellitus, use of insulin, and use of multiple (≥ 3) oral hypoglycemic agents.

**Supplementary Table 6. Impact of LDL-cholesterol change on the primary and secondary outcomes of new statin users**

|  | **n** | **Events** | **Duration (person-years)** | **Incidence** | **Hazard ratio (95% confidence interval)** | | | | ***P*-value^a^** |
| --- | --- | --- | --- | --- | --- | --- | --- | --- | --- |
|  |  |  |  |  | **Model 1** | **Model 2** | **Model 3** | **Model 4** |  |
| **Primary outcome** |  |  |  |  |  |  |  |  |  |
| Decreased LDL-cholesterol | 149,801 | 4,855 | 451,147 | 10.8 | 0.936 (0.897 - 0.976) | 0.970 (0.930 - 1.012) | 0.970 (0.928 - 1.013) | 0.992 (0.950 - 1.036) | 0.729 |
| Stable LDL-cholesterol | 108,611 | 3,909 | 337,930 | 11.6 | 1 (Reference) | 1 (Reference) | 1 (Reference) | 1 (Reference) |  |
| Increased LDL-cholesterol | 28,072 | 1,100 | 89,150 | 12.3 | 1.061 (0.992 - 1.134) | 1.150 (1.075 - 1.230) | 1.128 (1.054 - 1.206) | 1.119 (1.047 - 1.197) | <0.001 |
| **Secondary outcome** |  |  |  |  |  |  |  |  |  |
| Decreased LDL-cholesterol | 149,801 | 566 | 451,031 | 1.3 | 0.986 (0.870 - 1.118) | 1.023 (0.902 - 1.159) | 1.030 (0.904 - 1.173) | 1.050 (0.924 - 1.194) | 0.453 |
| Stable LDL-cholesterol | 108,611 | 432 | 337,857 | 1.3 | 1 (Reference) | 1 (Reference) | 1 (Reference) | 1 (Reference) |  |
| Increased LDL-cholesterol | 28,072 | 134 | 89,133 | 1.5 | 1.179 (0.964 - 1.420) | 1.248 (1.028 - 1.515) | 1.206 (0.992 - 1.466) | 1.196 (0.985 - 1.454) | 0.071 |

^a^*P*-value is described for the adjusted hazard ratio in Model 4.

Incidence is per 1,000 person*year follow-up.

LDL, low-density lipoprotein.

Model 1: Unadjusted.

Model 2: Adjusted for age and sex.

Model 3: Adjusted for age, sex, body mass index, income, smoking status, alcohol consumption status, regular exercise, hypertension, chronic kidney disease, cardiovascular disease, and previous LDL-cholesterol levels.

Model 4: Adjusted for age, sex, body mass index, income, smoking status, alcohol consumption status, regular exercise, hypertension, chronic kidney disease, cardiovascular disease, previous LDL-cholesterol levels, fasting glucose, duration of diabetes mellitus, use of insulin, and use of multiple (≥ 3) oral hypoglycemic agents.

**Supplementary Table 7. Impact of LDL-cholesterol change on the primary outcome**

|  | **n** | **Events** | **Duration (person-years)** | **Incidence** | **Hazard ratio (95% confidence interval)** | | | | ***P*-value^a^** |
| --- | --- | --- | --- | --- | --- | --- | --- | --- | --- |
|  |  |  |  |  | **Model 1** | **Model 2** | **Model 3** | **Model 4** |  |
| **Constant statin users** |  |  |  |  |  |  |  |  |  |
| ∆ LDL-cholesterol < –50 | 77,808 | 2,353 | 236,890 | 9.9 | 0.876 (0.836 - 0.917) | 1.002 (0.957 - 1.049) | 1.001 (0.955 - 1.050) | 1.029 (0.981 - 1.079) | 0.245 |
| –50 ≤ ∆ LDL-cholesterol < –20 | 48,625 | 1,615 | 147,903 | 10.9 | 0.963 (0.913 - 1.015) | 1.022 (0.969 - 1.078) | 1.015 (0.962 - 1.071) | 1.023 (0.970 - 1.079) | 0.409 |
| –20 ≤ ∆ LDL-cholesterol < 20 | 240,778 | 8,316 | 733,422 | 11.3 | 1 (Reference) | 1 (Reference) | 1 (Reference) | 1 (Reference) |  |
| 20 ≤ ∆ LDL-cholesterol < 50 | 30,274 | 1,057 | 93,954 | 11.3 | 0.988 (0.926 - 1.053) | 1.089 (1.022 - 1.161) | 1.063 (0.997 - 1.134) | 1.049 (0.984 - 1.118) | 0.144 |
| ∆ LDL-cholesterol ≥ 50 | 27,341 | 1,037 | 86,084 | 12.0 | 1.054 (0.988 - 1.124) | 1.247 (1.169 - 1.331) | 1.199 (1.124 - 1.279) | 1.177 (1.103 - 1.256) | <0.001 |
| **New statin users** |  |  |  |  |  |  |  |  |  |
| ∆LDL-cholesterol < –50 | 100,058 | 3,074 | 299,175 | 10.3 | 0.895 (0.853 - 0.938) | 0.945 (0.901 - 0.991) | 0.939 (0.893 - 0.987) | 0.967 (0.921 - 1.015) | 0.177 |
| –50 ≤ ∆ LDL-cholesterol < –20 | 49,743 | 1,781 | 151,972 | 11.7 | 1.016 (0.961 - 1.075) | 1.017 (0.961 - 1.075) | 1.017 (0.961 - 1.076) | 1.033 (0.977 - 1.093) | 0.258 |
| –20 ≤ ∆ LDL-cholesterol < 20 | 108,611 | 3,909 | 337,929 | 11.6 | 1 (Reference) | 1 (Reference) | 1 (Reference) | 1 (Reference) |  |
| 20 ≤ ∆ LDL-cholesterol < 50 | 15,807 | 592 | 49,733 | 11.9 | 1.026 (0.941 - 1.119) | 1.074 (0.985 - 1.171) | 1.071 (0.982 - 1.168) | 1.060 (0.972 - 1.156) | 0.189 |
| ∆ LDL-cholesterol ≥ 50 | 12,265 | 508 | 39,416 | 12.9 | 1.105 (1.007 - 1.212) | 1.253 (1.142 - 1.374) | 1.208 (1.100 - 1.325) | 1.202 (1.095 - 1.319) | <0.001 |
| **Non-statin users** |  |  |  |  |  |  |  |  |  |
| ∆ LDL-cholesterol < –50 | 27,627 | 1,578 | 87,331 | 18.1 | 1.296 (1.231 - 1.365) | 1.333 (1.266 - 1.404) | 1.487 (1.406 - 1.571) | 1.458 (1.379 - 1.541) | <0.001 |
| –50 ≤ ∆ LDL-cholesterol < –20 | 52,808 | 2,591 | 167,256 | 15.5 | 1.112 (1.067 - 1.159) | 1.122 (1.077 - 1.170) | 1.181 (1.131 - 1.232) | 1.172 (1.123 - 1.223) | <0.001 |
| –20 ≤ ∆ LDL-cholesterol < 20 | 374,751 | 16,538 | 1,187,480 | 13.9 | 1 (Reference) | 1 (Reference) | 1 (Reference) | 1 (Reference) |  |
| 20 ≤ ∆ LDL-cholesterol < 50 | 50,509 | 2,282 | 161,246 | 14.2 | 1.013 (0.970 - 1.059) | 1.090 (1.044 - 1.139) | 0.999 (0.955 - 1.045) | 1.001 (0.957 - 1.048) | 0.952 |
| ∆ LDL-cholesterol ≥ 50 | 26,565 | 1,321 | 85,564 | 15.4 | 1.103 (1.043 - 1.166) | 1.280 (1.210 - 1.353) | 1.065 (1.002 - 1.133) | 1.067 (1.004 - 1.134) | 0.037 |

^a^*P*-value is described for the adjusted hazard ratio in Model 4.

Incidence is per 1,000 person*year follow-up.

LDL, low-density lipoprotein.

Model 1: Unadjusted.

Model 2: Adjusted for age and sex.

Model 3: Adjusted for age, sex, body mass index, income, smoking status, alcohol consumption status, regular exercise, hypertension, chronic kidney disease, cardiovascular disease, and previous LDL-cholesterol levels.

Model 4: Adjusted for age, sex, body mass index, income, smoking status, alcohol consumption status, regular exercise, hypertension, chronic kidney disease, cardiovascular disease, previous LDL-cholesterol levels, fasting glucose, duration of diabetes mellitus, use of insulin, and use of multiple (≥ 3) oral hypoglycemic agents.

**Supplementary Table 8. Impact of LDL-cholesterol change on the secondary outcome**

|  | **n** | **Events** | **Duration (person-years)** | **Incidence** | **Hazard ratio (95% confidence interval)** | | | | ***P*-value^a^** |
| --- | --- | --- | --- | --- | --- | --- | --- | --- | --- |
|  |  |  |  |  | **Model 1** | **Model 2** | **Model 3** | **Model 4** |  |
| **Constant statin users** |  |  |  |  |  |  |  |  |  |
| ∆ LDL-cholesterol < –50 | 77,808 | 319 | 236,826 | 1.3 | 0.897 (0.792 - 1.016) | 1.011 (0.892 - 1.145) | 1.037 (0.903 - 1.192) | 1.078 (0.943 - 1.232) | 0.270 |
| –50 ≤ ∆ LDL-cholesterol < –20 | 48,625 | 202 | 147,860 | 1.4 | 0.910 (0.783 - 1.058) | 0.956 (0.823 - 1.111) | 0.959 (0.824 - 1.116) | 0.968 (0.832 - 1.126) | 0.670 |
| –20 ≤ ∆ LDL-cholesterol < 20 | 240,778 | 1,100 | 733,218 | 1.5 | 1 (Reference) | 1 (Reference) | 1 (Reference) | 1 (Reference) |  |
| 20 ≤ ∆ LDL-cholesterol < 50 | 30,274 | 139 | 93,918 | 1.5 | 0.983 (0.824 - 1.173) | 1.057 (0.886 - 1.261) | 1.018 (0.853 - 1.215) | 1.007 (0.844 - 1.201) | 0.940 |
| ∆ LDL-cholesterol ≥ 50 | 27,341 | 130 | 86,065 | 1.5 | 1.001 (0.834 - 1.200) | 1.145 (0.954 - 1.373) | 1.082 (0.902 - 1.299) | 1.072 (0.893 - 1.287) | 0.456 |
| **New statin users** |  |  |  |  |  |  |  |  |  |
| ∆LDL-cholesterol < –50 | 100,058 | 372 | 299,104 | 1.2 | 0.979 (0.852 - 1.124) | 1.034 (0.900 - 1.188) | 1.038 (0.896 - 1.202) | 1.067 (0.923 - 1.232) | 0.381 |
| –50 ≤ ∆ LDL-cholesterol < –20 | 49,743 | 194 | 151,927 | 1.3 | 1.001 (0.845 - 1.186) | 1.002 (0.846 - 1.187) | 1.011 (0.853 - 1.199) | 1.019 (0.860 - 1.208) | 0.827 |
| –20 ≤ ∆ LDL-cholesterol < 20 | 108,611 | 432 | 337,857 | 1.3 | 1 (Reference) | 1 (Reference) | 1 (Reference) | 1 (Reference) |  |
| 20 ≤ ∆ LDL-cholesterol < 50 | 15,807 | 74 | 49,725 | 1.5 | 1.161 (0.907 - 1.485) | 1.203 (0.940 - 1.539) | 1.189 (0.929 - 1.522) | 1.178 (0.920 - 1.507) | 0.195 |
| ∆ LDL-cholesterol ≥ 50 | 12,265 | 60 | 39,409 | 1.5 | 1.181 (0.901 - 1.547) | 1.307 (0.998 - 1.713) | 1.237 (0.942 - 1.624) | 1.235 (0.941 - 1.620) | 0.128 |
| **Non-statin users** |  |  |  |  |  |  |  |  |  |
| ∆ LDL-cholesterol < –50 | 27,627 | 189 | 87,282 | 2.2 | 1.392 (1.198 - 1.616) | 1.446 (1.245 - 1.679) | 1.416 (1.213 - 1.654) | 1.427 (1.224 - 1.664) | <.001 |
| –50 ≤ ∆ LDL-cholesterol < –20 | 52,808 | 286 | 167,216 | 1.7 | 1.099 (0.971 - 1.245) | 1.114 (0.984 - 1.262) | 1.107 (0.977 - 1.255) | 1.116 (0.985 - 1.265) | 0.084 |
| –20 ≤ ∆ LDL-cholesterol < 20 | 374,751 | 1,847 | 1,187,193 | 1.6 | 1 (Reference) | 1 (Reference) | 1 (Reference) | 1 (Reference) |  |
| 20 ≤ ∆ LDL-cholesterol < 50 | 50,509 | 278 | 161,206 | 1.7 | 1.106 (0.975 - 1.255) | 1.178 (1.039 - 1.337) | 1.112 (0.979 - 1.264) | 1.098 (0.966 - 1.247) | 0.153 |
| ∆ LDL-cholesterol ≥ 50 | 26,565 | 150 | 85,542 | 1.8 | 1.122 (0.950 - 1.326) | 1.273 (1.078 - 1.504) | 1.107 (0.930 - 1.317) | 1.080 (0.908 - 1.284) | 0.387 |

^a^*P*-value is described for the adjusted hazard ratio in Model 4.

Incidence is per 1,000 person*year follow-up.

LDL, low-density lipoprotein.

Model 1: Unadjusted.

Model 2: Adjusted for age and sex.

Model 3: Adjusted for age, sex, body mass index, income, smoking status, alcohol consumption status, regular exercise, hypertension, chronic kidney disease, cardiovascular disease, and previous LDL-cholesterol levels.

Model 4: Adjusted for age, sex, body mass index, income, smoking status, alcohol consumption status, regular exercise, hypertension, chronic kidney disease, cardiovascular disease, previous LDL-cholesterol levels, fasting glucose, duration of diabetes mellitus, use of insulin, and use of multiple (≥ 3) oral hypoglycemic agents.
